# Investigation of the Influence of the Extraction System and Seasonality on the Pharmacological Potential of *Eugenia punicifolia* Leaves

**DOI:** 10.3390/molecules30030713

**Published:** 2025-02-05

**Authors:** Kidney O. G. Neves, Samuel O. Silva, Marinildo S. Cruz, Josiana Moreira Mar, Jaqueline A. Bezerra, Edgar A. Sanches, Natasha Marques Cassani, Giovanna A. Antoniucci, Ana Carolina Gomes Jardim, Francisco C. M. Chaves, Leonard D. R. Acho, Emersom S. Lima, Marcos B. Machado, Alan D. C. Santos

**Affiliations:** 1 Núcleo de Estudos Químicos de Micromoléculas da Amazônia—NEQUIMA, Universidade Federal do Amazonas, Manaus 69067-005, AM, Brazil; kidneydeoliveira@ufam.edu.br (K.O.G.N.); samuel-oliveira.silva@ufam.edu.br (S.O.S.); marinildo.cruz@ufam.edu.br (M.S.C.); 2 Laboratório de Polímeros Nanoestruturados (NANOPOL), Departamento de Física de Materiais, Universidade Federal do Amazonas, Manaus 69067-005, AM, Brazil; josimoreira@ufam.edu.br (J.M.M.); jaqueline.araujo@ifam.edu.br (J.A.B.); sanchesufam@ufam.edu.br (E.A.S.); 3 Laboratory of Antiviral Research, Federal University of Uberlândia, Uberlândia 38405-302, MG, Brazil; natashacassani@ufu.br (N.M.C.); giovanna.antoniucci@ufu.br (G.A.A.); jardim@ufu.br (A.C.G.J.); 4 Empresa Brasileira de Pesquisa Agropecuária—Embrapa Amazônia Ocidental, Manaus 69010-970, AM, Brazil; celio.chaves@embrapa.br; 5 Laboratório de Atividade Biológica, Faculdade de Ciências Farmacêuticas, Universidade Federal do Amazonas, Manaus 69067-005, AM, Brazil; leonard.rosale@ufam.edu.br (L.D.R.A.); eslima@ufam.edu.br (E.S.L.); 6 Núcleo de Pesquisa de Produtos Naturais, Universidade Federal de Santa Maria, Santa Maria 97105-900, RS, Brazil

**Keywords:** pedra-ume-caá, medicinal plant, solvent extraction, antioxidant, antiglycation, antiviral, Zika virus, phenolic compounds, NMR spectroscopy, CCA

## Abstract

The chemical complexity of natural products, such as *Eugenia punicifolia* (Kunth) DC. plant, presents a challenge when extracting and identifying bioactive compounds. This study investigates the impact of different extraction systems and seasonal variations on the chemical profile and pharmacological potential of *E. punicifolia* leaves using NMR spectroscopy for chemical analysis and canonical correlation analysis (CCA) for bioactivity correlation. Extracts obtained with methanol (M), ethanol (E), methanol/ethanol (1:1, ME), and methanol/ethanol/water (3:1:1, MEW) were analyzed for antioxidant, antiglycation, and antiviral activities. Quantitative ¹H NMR, combined with the PULCON method, was used to quantify phenolic compounds such as quercetin, myricetin, catechin, and gallic acid. The results showed that the MEW extract obtained in the rainy season exhibited the highest antioxidant and antiglycation activities, with a greater than 93% of advanced-glycation end-products (AGEs) inhibition capacity. Furthermore, our results showed that all the extracts were able to inhibit over 94% of the Zika virus (ZIKV) infection in Vero E6 cells. The CCA established strong correlations between the phenolic compounds and bioactivities, identifying gallic acid, catechin, quercetin, and myricetin as key chemical markers. This study demonstrates the importance of selecting appropriate extraction systems and considering seasonality to optimize the pharmacological potential of *E. punicifolia* leaves and highlights the efficacy of NMR in linking chemical composition with bioactivities.

## 1. Introduction

*Eugenia punicifolia* (Kunth) DC., a species that is both native and endemic to Brazil, is widely distributed throughout the Amazon region. Commonly known as a “vegetable insulin”, this plant is part of a group of species known as pedra-ume-caá, which are traditionally used in herbal medicine [1,2,3]. Research on this matrix has demonstrated that its leaves contain barbinervic acid, a compound with vasodilatory effects. This compound shows significant potential as a template for developing new molecules to treat cardiovascular diseases [4]. Basting et al. (2014) demonstrated that the hydroalcoholic extract from the leaves has significant antinociceptive and anti-inflammatory effects, which may be related to the inhibition of the glutamatergic system, nitric oxide synthesis, and the phosphorylation of p38α MAPK [5]. Furthermore, Oliveira et al. (2022), Sales et al. (2014), and Ramos et al. (2019) showed that the leaves and fruits of this species exhibit antioxidant and antiglycation potential, as well as a chemical composition rich in flavonoids and organic acids with various pharmacological properties, particularly for the treatment of diabetes mellitus [2,6,7]. *E. punicifolia* is frequently marketed in the Amazon for this purpose. Its widespread use in this region has driven scientific interest in exploring its pharmacological potential, especially its ability to manage blood glucose levels in diabetic patients [2,6,7,8,9].

In general, natural products are chemically complex and contain a wide variety of bioactive compounds, including alkaloids, flavonoids, terpenes, lignoids, and phenolic acids, each contributing to the plant’s overall pharmacological activity. This complexity, coupled with the typically low concentrations of these bioactive compounds, poses significant challenges in the chemical analysis of such matrices, making the choice of extraction methodology crucial. Extraction serves as the initial step to isolate the desired bioactive compounds from the raw material and can provide a clear snapshot of the plant’s chemical profile, while the type of extraction used can maximize both the yield and selectivity of active principles [10].

This matter has been exemplified in the work published by Neves et al. (2004), who investigated the influence of seasonal variation (dry, rainy, and transition periods) on the ¹H NMR chemical profiles and antioxidant potential of *E. punicifolia* leaf extracts obtained with dimethyl sulfoxide (DMSO) [11]. Although variations in the chemical profiles and antioxidant activities were observed between the seasons, the ¹H NMR data did not provide sufficient insight into the correlation between secondary metabolites and bioactivity, since DMSO favored the extraction of primary metabolites. This limitation highlights the need to explore alternative extraction methods to better establish the link between secondary metabolites and bioactivity.

Although several studies have reported extraction methods for analyzing the chemical composition of *E. punicifolia*, only a few have investigated or optimized these processes to assess their impact on biological activity [9,12,13]. Among them, the work of Santos et al. (2020) stands out for its focus on optimizing the recovery of phenolic compounds with enhanced antioxidant and antiproliferative activities. Using a multivariate analytical approach, they developed an optimized extraction method for *E. punicifolia* leaves. Among the solvents tested (ethanol, methanol, and water), ethanolic extracts yielded the highest phenolic content, exhibited the strongest antioxidant activity, and demonstrated moderate antiproliferative activity against HEp-2 cells [9]. Santos et al.’s (2020) study provides a solid foundation for research on extraction methods for *E. punicifolia* and served as the starting point for our current investigation.

Nuclear magnetic resonance spectroscopy (NMR) has played an important role in tracking the qualitative and quantitative profiles of metabolites in plants, offering relevant insight into their complex chemical compositions [14,15,16]. This technique is essential for establishing correlations between the chemical profiles of plant extracts and their biological activities, often referred to as spectrum–effect relationships [17,18,19]. By providing detailed molecular information, NMR allows researchers to link specific metabolites to pharmacological effects, aiding in the identification of key bioactive compounds and optimizing the extraction methods for targeted applications.

In this context, the present study aimed to identify the most effective extraction solvent for correlating the quantitative chemical profiles, obtained through NMR spectroscopy, with the pharmacological potential (antioxidant, antiglycation, and antiviral) of *E. punicifolia* leaves collected during different seasonal periods.

## 2. Results and Discussion

### 2.1. The Performance of the Extraction Systems Tested

Water, methanol, ethanol, their mixtures, and aqueous acetone solutions are commonly used for the extraction of phenolic compounds [9]. However, the wide diversity of phenolics in plants poses a challenge to the standardization of extraction methods, particularly in selecting the most suitable solvent [9,11].

The efficiency of the extraction system was evaluated by calculating the mean and standard deviation of yield values obtained in triplicate (Table 1). The ternary mixture MEW (methanol/ethanol/water) produced the highest yields, with overall standard deviations ranging from 7.31% to 13.46%. The method’s reproducibility was assessed using an ANOVA of the mean yields, considering both the extraction solvent and the collection period. This analysis demonstrated satisfactory reproducibility, as no significant statistical differences in extraction yields were observed across samples collected in different periods as a function of the extraction solvent—except for the samples collected during the dry season and extracted with methanol. This indicates that the extraction efficiency is slightly affected by the season, though it confirms that, overall, the extraction systems are suitable for obtaining *E. punicifolia* leaf extracts.

### 2.2. Bioactivities of the E. punicifolia Leaf Extracts

#### 2.2.1. Cytotoxicity of *Eugenia punicifolia* Leaf Extracts

Cytotoxicity, or assessing cell viability, is a critical step in evaluating the antiviral potential of plant extracts and substances, as it indicates the ability of a substance or extract to cause cellular damage or death [20]. In this study, to investigate potential cytotoxicity, Vero E6 cells (kidney tissue derived from a normal, adult African green monkey) were each treated with *E. punicifolia* extracts at the concentrations of 50, 10, and 2 μg mL^−1^ for 72 h. Then, cell viability was assessed via an MTT assay. DMSO (0.1%) was used as the untreated control. Analyzing the effects of the tested extracts on cell viability, we found that the treatment of Vero E6 cells with extracts at the concentration of 50 μg mL^−1^ presented cell viability over 90% (Figure S1).

#### 2.2.2. Anti-ZIKV Activity

**Figure 1:**
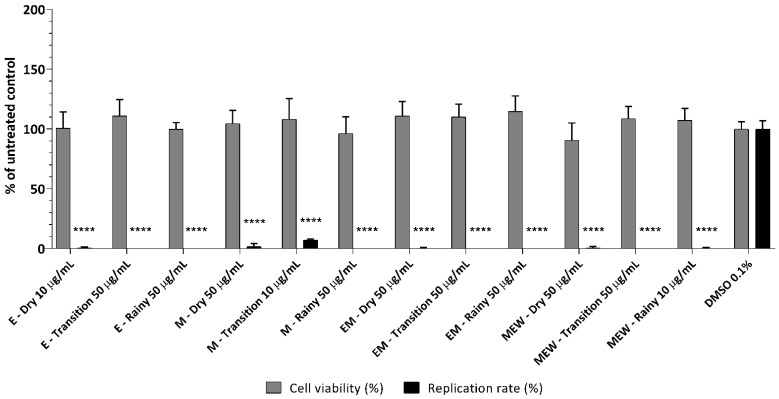
Effect of extracts of *E*. *punicifolia* leaves on viability of Vero E6 cells and ZIKV infectivity. Vero E6 cells were infected with ZIKV_PE243_ at an MOI of 0.01 in the presence or absence of each extract at the highest non-cytotoxic concentration for 72 h. Then, the cells were fixed, and an immunofluorescence assay was performed. Focus-forming units (FFUs) were counted. The viability assay was performed in parallel by treating Vero E6 cells with each compound at the previously established non-cytotoxic concentration, and absorbance was measured (560 nm). DMSO (0.1%) was used as the untreated control. The mean values of two independent experiments, each performed in triplicate, including the standard error of the mean, are shown. *P* values < 0.05 were considered significant. (****) *p* < 0.0001. Extract acronyms: MEW—methanol/ethanol/water; M—methanol; EM—ethanol/methanol; and E—ethanol.

Due to its traditional use in the treatment of type 2 diabetes mellitus, research on *E. punicifolia* has primarily focused on evaluating its antiglycation potential [2,6,7]. However, given the species’ diverse chemical composition and its use in regions frequently affected by viruses, including ZIKV, it is crucial to investigate its potential antiviral properties. In this study, the anti-ZIKV activity of the extracts was assessed using Vero E6 cells infected with ZIKV_PE243_ in the presence or absence of the extracts for 72 h. The results showed that the extracts at the established non-cytotoxic concentration were able to inhibit up to 100% of ZIKV infection, with the minimum inhibitory rate of 94.8% under treatment with the M-Transition extract (Figure 1). This is the first study to demonstrate that *E. punicifolia* leaf extracts can inhibit ZIKV replication, which enhances the value of this plant species. However, additional assays should be performed to better understand the mechanism of action of these extracts and their cytotoxic effects, since they were tested in a general MTT and infection assay. The observed reduction in viral replication could be a result of either a virucidal activity or inhibition of viral replication cycle within the host cells.

#### 2.2.3. Antioxidant Activity via DPPH and ABTS Assays

DPPH and ABTS assays provide a low-cost and efficient method for determining the oxidation-inhibiting capacity of plant-derived substances and extracts [21,22]. As such, these assays can be used as probes to assess the impact of external factors on the chemical composition of plant matrices [23,24].

The DPPH and ABTS assays demonstrated that, regardless of the extraction system used, samples collected during the rainy season exhibited the strongest antioxidant responses (Table 2). Among the extraction systems, EM and MEW yielded the best results; however, MEW showed an antioxidant response of 8% to 16%, which is higher than that of the samples extracted with EM. The Pearson correlation for the antioxidant assays was 0.923 (*p* < 0.05), indicating a strong correlation between the assays and confirming the antioxidant potential of the samples collected during the rainy season and extracted using the MEW system.

#### 2.2.4. Antiglycation Activity Assay: Non-Oxidative Pathway

Type 2 diabetes mellitus, characterized by persistent hyperglycemia, leads to non-enzymatic glycation reactions with proteins and lipids, marking the initial stage in the formation of advanced-glycation end-products (AGEs) [25]. Since AGEs play a critical role in diabetic complications, identifying plant matrices rich in compounds that can inhibit the glycation process has become a promising and effective approach. In this study, the ability of various *E. punicifolia* leaf extracts to inhibit AGE formation via the non-oxidative pathway was evaluated (Table 3).

The results demonstrate that all the extracts exhibited an inhibition potential greater than 75%. Furthermore, with the exception of the methanol extracts, significant differences in inhibition capacities were observed between the samples collected during the dry and rainy seasons. The rainy season samples showed up to 15% higher inhibition compared to those collected in the dry season. This finding confirms that the collection period is a critical factor when evaluating the antiglycation potential of *E. punicifolia* leaves.

### 2.3. Identification of Phenolic Compounds in E. punicifolia Leaf Extracts

**Figure 2:**
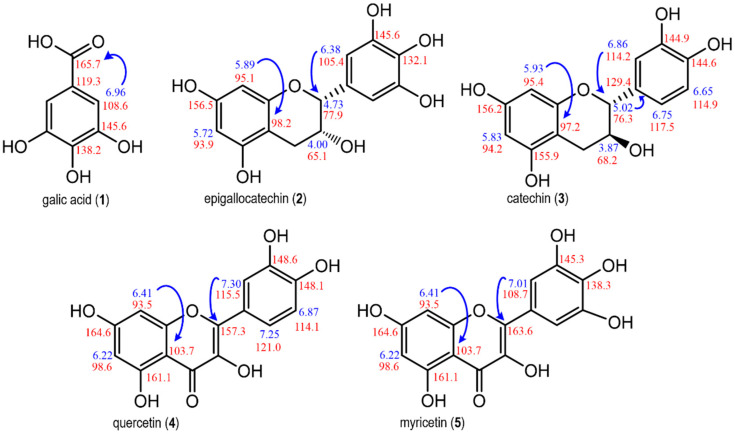
Compounds identified by NMR spectroscopy in different *E. punicifolia* leaf extracts. Blue arrows represent key correlations observed in the long-range HMBC correlation plot.

The four extracts (E, M, EM, and MEW) of *E. punicifolia* leaves were analyzed via NMR spectroscopy, which led to the identification of gallic acid (**1**) and four flavonoids: epigallocatechin (**2**), catechin (**3**), quercetin (**4**), and myricetin (**5**) (Figures S2–S6). Gallic acid was identified by correlating the signal at δ 6.95 (H-2 and H-6, s) with the signals observed at 165.7 (COOH), 108.6 (C-2 and C-6), 145.6 (C-3 and C-5), and 138.2 (C-4) in the long-range ^1^H-^13^C HMBC correlation map [26]. While the signals at δ 5.89 (H-8, d, 2.3 Hz), δ 5.72 (H-6, d, 2.3 Hz), and δ 6.38 (H-2′ and H-5′, s) were attributed to rings A and B of epigallocatechin, along with the singlet at δ 4.73 (H-2, s) and the multiplet at δ 4.00 (H-3, m) referring to its C ring [27]. The presence of catechin in the extracts was supported by signals at δ 5.83 (H-6, d, 2.3 Hz), δ 5.93 (H-8, d, 2.3 Hz), δ 6.65 (H-5′, d, 8.1 Hz), δ 6.75 (H-6′, dd, 2.3 Hz and 8.1 Hz), and δ 6.86 (H-2′, d, 2.3 Hz), corresponding to rings A and B, as well as the doublet at δ 5.02 (H-2, d, 1.8 Hz) referring to the C ring [28]. Quercetin and myricetin both exhibited signals at δ 6.41 (H-8, d, 2.1 Hz) and δ 6.22 (H-6, d, 2.1 Hz) belonging to ring A; however, in ring B, myricetin presented a signal at δ 7.01 (H-2′ and H-5′, s), while quercetin displayed signals at δ 7.30 (H-2′, d, 2.1 Hz), δ 7.25 (H-6′, dd, 2.1 and 8.3 Hz), and δ 6.87 (H-5′, d, 8.3 Hz) [28]. The connection of the B ring with the C ring of the flavonoids was assigned based on the correlations observed in the HMBC correlation plot, as shown in Figure 2.

### 2.4. qNMR of Phenolic Compounds by PULCON

PULCON (Pulse Length-Based Concentration Determination) is a powerful NMR method for quantifying compounds in complex mixtures without requiring specific standards for the compounds of interest [29,30]. The method is based on the principle of reciprocity, which correlates the absolute intensities in two one-dimensional (1D) NMR spectra [31,32]. Using PULCON, the ^1^H NMR signals corresponding to gallic acid (δ 6.96, s), epigallocatechin (δ 5.89, d, 2.3 Hz), catechin (δ 5.93, d, 2.3 Hz), quercetin (δ 7.30, d, 2.1 Hz), and myricetin (δ 7.01, s) were quantified in the different extracts of *E. punicifolia* leaves (Figures S7–S12), as summarized in Table 4. Overall, the concentrations of compounds in the *E. punicifolia* extracts can be categorized into three groups: (I) catechin as the most abundant phenolic compound, (II) gallic acid and epigallocatechin at intermediate concentrations and (III) quercetin and myricetin as the least abundant. The specific ranking within groups II and III depended on the extraction solvent used and the period considered.

Table 4 also presents the NMR-quantified phenolic compounds, representing the sum of all the identified and quantified phenolic compounds. These values clearly demonstrate the influence of the extraction procedure on the selectivity of phenolic compounds, with MEW being the most selective solvent and E the least selective. Notably, the total phenolic content suggested a dependency between the extraction solvent’s selectivity and the periods (dry, transition, and rainy). For the MEW samples, no significant statistical differences in total phenolics were observed among the periods. In contrast, extractions with E showed significant seasonal variation.

The phenolic content provided by PULCON can be a valuable tool in understanding the relationship between the chemical composition and biological activity of *E. punicifolia* leaf extracts, especially since their phenolic profiles are qualitatively similar. In such cases, differences in biological activities may be linked to variations in phenolic content.

### 2.5. Chemical Composition and Bioactivities of the E. punicifolia Leaf Extracts: Searching for Correlations

**Figure 3:**
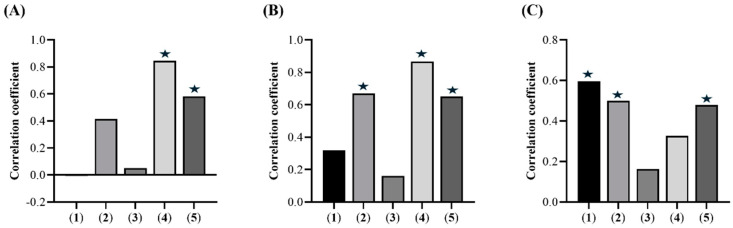
Canonical correlation analysis. Pearson correlation coefficients (**★**, r > 0.50) between the concentrations determined using ^1^H NMR and the scavenging capacity of the DPPH radical (**A**), the ABTS radical cation (**B**), and AGE formation inhibition (**C**) **1**—gallic acid, **2**—epigallocatechin, **3**—catechin, **4**—quercetin, and **5**—myricetin.

The correlation between the compounds quantified via ^1^H NMR and the pharmacological potential of *E. punicifolia* leaf extracts (Figure 3) was assessed using a canonical correlation analysis (CCA), a multivariate statistical technique that identifies and quantifies the relationship between two sets of variables [33]. Pearson’s correlation coefficient was applied as the index to evaluate the strength or existence of this correlation (Table S1) [34,35].

An analysis of the Pearson correlation values with the antioxidant assays revealed that 2-epigallocatechin (r = 0.67), quercetin (r = 0.87), and myricetin (r = 0.65) exhibit a moderately positive correlation with the ABTS radical cation scavenging capacity. Additionally, only quercetin (r = 0.85) and myricetin (r = 0.58) were found to correlate with the DPPH radical scavenging capacity (Figure 3). Several studies on these flavonoids have demonstrated their ability to scavenge free radicals in the body [36,37].

In 2024, a study on the antioxidant potential of DMSO extracts of *E. punicifolia* leaves revealed that samples collected during the dry period exhibited the strongest antioxidant response [11]. This supports the notion that the extraction system is a key factor since it influences not only the antioxidant activity but also any pharmacological activity of plant matrices. For *E. punicifolia*, DMSO extraction primarily favored carbohydrates and fatty acids, whereas the extraction systems described in this study favored flavonoid compounds. Among the identified flavonoids, myricetin was notably absent in the DMSO-extracted samples. Given myricetin’s high antioxidant potential, its presence likely contributes to the differences in antioxidant capacity observed between the various extraction systems [38]. These findings highlight the need to establish a standardized extraction protocol to ensure the chemical profile is consistent and serves as a reliable basis for comparison between studies on plant matrices.

In the AGE formation inhibition assay, the Pearson’ correlation analysis revealed that only gallic acid (r = 0.60), 2-epigallocatechin (r = 0.50), and myricetin (r = 0.48) showed a moderately positive correlation (Figure 3). Studies on these compounds demonstrate their crucial role in inhibiting the early stages of glycation, thus preventing the formation of AGEs [39]. Furthermore, Wang et al. (2024) reported that myricetin and its derivatives can completely inhibit products generated by non-enzymatic glycation reactions [40]. Along with their association with the antiglycation activity of *E*. *punicifolia* leaves, gallic acid, catechin, and myricetin have also been suggested by Oliveira et al. (2024) as key chemical markers for species of *Pedra-ume-caá* [2].

Given that all *E. punicifolia* extracts demonstrated variable cytotoxicity in Vero E6 cells and a ZIKV infection inhibition capacity greater than 95%, this response is likely attributed to the phenolic compounds identified and quantified in the extracts [41,42]. Some components of the extracts enhanced cell viability at certain concentrations, and this might be due to the fact that plant-derived polyphenols, including flavonoids, exhibit antioxidant properties that support cell survival and growth under certain conditions [43,44]. Myricetin has shown concentration-dependent protective effects, enhancing cellular repair and proliferation at optimal levels [44]. Likewise, quercetin has been linked to improved mitochondrial function and increased metabolic activity, aiding cell viability, especially under stress, which can further be seen by different effects in different concentrations [45].

Regarding the antiviral properties, a study by Lim et al. (2017) showed that myricetin can inhibit 88% of ZIKV_NS2B-NS3_ activity [46]. Similarly, an in vitro study by Zou et al. (2020) found that, at a concentration of 1.0 mM, myricetin and quercetin can inhibit up to 80% of ZIKV_NS1_ infection, as also found by Ramos et al. (2022), with quercetin inhibiting ZIKV_NS5_ RNA-dependent RNA polymerase (RdRp) with IC_50_ values of 0.5 μM [47]. This inhibitory capacity has been linked to the presence of hydroxyl groups on ring B of these compounds [48].

Thus, the canonical correlation analysis, with the Pearson’s correlation coefficient as an index, proved to be an effective approach, allowing for the identification of gallic acid, catechin, quercetin, and myricetin as chemical indicators for monitoring the antioxidant, antiglycation, and antiviral activities of *E. punicifolia* leaf extracts in relation to the collection period.

## 3. Materials and Methods

### 3.1. Materials

The methanol (HPLC) and absolute ethanol (99.5% PA) used for plant material extraction were purchased from Sigma-Aldrich (St. Louis, MO, USA). The deuterated dimethyl sulfoxide (DMSO-*d*6, 99.9%) with tetramethylsilane (TMS, 0.05% *V*/*V*) for NMR analyses was obtained from Cambridge Isotope Laboratories Inc. (Andover, MA, USA). Dimethyl terephthalate (DMT), a certified reference material, was provided by the Division of Chemical and Thermal Metrology (Inmetro, Rio de Janeiro, Brazil), under certification number DIMCI 1507/2019 (certified purity: 99.988 ± 0.060%). The reagents used in antioxidant assays, including 6-hydroxy-2,5,7,8-tetramethylchroman-2-carboxylic acid (Trolox), 2,2-diphenyl-1-picrylhydrazyl (DPPH^•^), and 2,2′-azino-bis(3-ethylbenzothiazoline-6-sulfonic acid) and ammonium salt (ABTS^•+^), were obtained from Sigma-Aldrich (St. Louis, MO, USA). Similarly, albumin, fructose and sodium azide used in the antiglycation assay were sourced from Sigma-Aldrich (St. Louis, MO, USA). For the cytotoxic and antiviral assay, Dulbecco’s Modified Eagle’s Medium (DMEM) and 3-(4,5-dimethylthiazol-2-yl)-2,5-diphenyltetrazolium bromide (MTT) were obtained from Sigma-Aldrich (St. Louis, MO, USA), while penicillin, streptomycin, non-essential amino acids, and fetal bovine serum were sourced from Gibco Life Technologies (Carlsbad, CA, USA). The ZIKV rabbit anti-NS3 primary antibody was kindly provided by Professor Andres Merits [49]. Goat anti-rabbit IgG Alexa Fluor 488 was sourced from Invitrogen (Waltham, MA, USA).

### 3.2. Plant Material

The leaves of *E*. *punicifolia* were collected during different months [August 2021 (dry period), December 2021 (transition period), and March 2022 (rainy period)] at the Brazilian Agricultural Research Corporation—Embrapa Amazônia Ocidental—located along Rodovia AM-010, Km 29 (2°53′23″ S 59°58′26″ W). Access to genetic heritage was registered (A82BD35) in the National System of Management of Genetic Heritage and Associated Traditional Knowledge (SisGen). From a plantation of 150 individuals, 15 trees were randomly selected, and leaves were gathered from various heights to ensure a representative sample (11 leaves each from the lower, middle, and upper parts of the trees). The collected plant material was dried at room temperature for 24 h, followed by 48 h in a forced air circulation oven at 40 °C. After drying, each sample was subjected to the cold maceration process with liquid nitrogen, weighed, and then stored in a freezer at −80 °C until the extraction procedure.

### 3.3. Extraction Procedure

The extraction systems used were selected based on the methodology described by Santos et al. (2020) [9]. For the extractions, 1.0 g of the sample mixture from each collection was subjected, in triplicate, to four different extraction systems: [1: methanol (100%)—M; 2: ethanol (100%)—E; 3: ethanol (50%)/methanol (50%)—EM; and 4: methanol (60%)/ethanol (20%)/water (20%)—MEW]. Each extraction was performed four times and sonicated in an ultrasonic bath for 15 min, followed by centrifugation at 4000 rpm for 10 min (4226× *g*). The supernatant was then separated and dried using nitrogen gas. Statistical analysis of the extraction yields was conducted using Minitab 18.1, employing the analysis of variance (ANOVA) with the Tukey’s test and a significance level of 95% [50].

### 3.4. Acquisition and Processing of NMR Data

Twenty milligrams (20.0 mg) of each *E*. *punicifolia* leaf extract was solubilized in 520 μL of DMSO-*d*_6_, sonicated in an ultrasonic bath for 10 min, and then transferred to a 5 mm NMR tube. NMR spectra were acquired using a Bruker Avance IIIHD NMR spectrometer (Bruker, Billerica, MA, USA), operating at 11.7 T (500 MHz for ^1^H) and equipped with a 5 mm BBFO Plus SmartProbe™ with a Z-axis gradient. The pulse sequences used were obtained from the Bruker database. The zgpr pulse sequence was used, with the following acquisition parameters: time domain (TD) data points of 32k, spectral width (SW) of 8 kHz, acquisition time (AQ) of 1.64 s, receiver gain (RG) of 90.5, number of scans (NS) equal to 32, dummy scans of 2, FID resolution of 0.30 Hz, central frequency (O1) set to 1667.48 Hz, and suppression power (PLW9) of 8.6289e^−005^. The P1 value was automatically calculated for each sample using the pulsecal sn command. The D1 values for signals corresponding to the aromatic compounds were calculated using Equation (1). The longitudinal relaxation constant (T1) was determined using an inversion–recovery experiment (t1ir) pulse sequence, and the highest T1 value was used to determine the D1 value (15 s) for the sample acquisition.

D1 = 7 × T1 − AQ(1)

The dimethyl terephthalate (DMT) was prepared in triplicate at a concentration of 20.12 mM in DMSO-*d*_6_ (D, 99.9%) with TMS (0.05% *v*/*v*) as an internal reference standard (0.00 ppm). For the quantitative ^1^H NMR spectrum, the 90° pulse of DMT (10.65 μs) was calculated for the signal at δ 8.10 (s, 4H) using the 90° pulse experiment (zg). The determination of T1 was carried out using the t1ir1d experiment for the signal at δ 8.10. After obtaining the T1 value, D1 (16.62 s) was estimated using Equation (1), for which the acquisition time (AQ) was set to 1.64 s. Except for the P1 and D1 parameters, the same acquisition parameters used for the quantitative spectra of the extracts were applied to the acquisition of DMT.

Phase and baseline corrections of the spectra were performed manually using TopSpin 3.6.3 software. The chemical shift (in ppm) of the ^1^H NMR spectra was referenced to the methyl signal of tetramethylsilane at δ_H_ 0.00, and the coupling constants (J) were recorded in Hz. HSQC and HMBC NMR experiments were conducted to verify the absence of signal overlap with the signals of interest, with the ^1^H-^13^C correlations acquired using coupling constants J (H, C—single bond) and J (H, C—long-range) of 145 and 8 Hz, respectively. Signal integration was performed manually, and the quantification of phenolic compounds using the PULCON method was carried out with the ERETIC2 (electronic reference to access in vivo concentrations) tool in TopSpin 3.6.3 software [51,52].

### 3.5. Cell Culture

The Vero E6 cells (kidney tissue derived from a normal adult African green monkey, ATCC E6/CRL-1586) were cultured in Dulbecco’s modified Eagle’s medium supplemented with 100 U mL^−1^ penicillin, 100 mg mL^−1^ streptomycin, 1% (*v*/*v*) non-essential amino acids, and 10% (*v*/*v*) fetal bovine serum at 37 °C in a humidified 5% CO_2_ incubator.

### 3.6. Cell Viability Assay

Cell viability was measured via the MTT [3-(4,5-dimethylthiazol-2-yl)-2,5-diphenyl tetrazolium bromide] method. The Vero E6 cells were seeded in 96-well plates at a density of 5 × 10^3^ cells per well and incubated overnight at 37 °C in a humidified 5% CO_2_ incubator. The drug-containing medium at the concentrations of 50, 10, and 2.0 μg mL^−1^ was added to the cell culture for 72 h at 37 °C. Then, the medium was removed, and a solution containing MTT at the final concentration of 1 mg mL^−1^ was added to each well and incubated for 30 min at 37 °C in a humidified 5% CO_2_ incubator, after which media were replaced with 100 μL of DMSO to solubilize the formazan crystals. Absorbance was measured using the optical density (OD) of each well at 560 nm, using a GloMax^®^ microplate reader (Promega, Madison, WI, USA). Cell viability was calculated according to the equation (T/C) × 100%, where T and C represent the mean optical density of the treated group and vehicle control group, respectively.

### 3.7. Antiviral Assay—ZIKV

A wild-type ZIKV isolate from a clinical sample of a patient in Brazil (ZIKV_PE243_) was amplified employing infected Vero E6 cells in 75 cm² flask for 3 days [53]. Then, the viral supernatant was collected and stored at −80 °C. To determine viral titers, 5 × 10^3^ Vero E6 cells were seeded in each of the 96-well plates 24 h prior to the infection. Cells were infected with 10-fold serial dilution of ZIKV_PE243_ and incubated for 72 h in a humidified 5% CO_2_ incubator at 37 °C. Following this, the cells were fixed with 4% formaldehyde, washed with phosphate-buffered saline (PBS), and a blocking buffer (BB) was added containing 0.10% Triton X-100, 0.20% bovine albumin, and PBS for 30 min. An immunofluorescence assay was performed as previously described [54]. In summary, the cells were incubated with the ZIKV anti-NS3 primary antibody, followed by a second incubation with the goat anti-rabbit IgG Alexa Fluor 488. Images were analyzed using EVOs Cell Imaging Systems Fluorescence Microscopy (Thermo Fisher Scientific), and the foci of infection were counted and measured as focus-forming units (FFUs mL^−1^) for viral titer determination.

To assess the antiviral activity of each extract, the Vero E6 cells were seeded at a density of 5 × 10^3^ cells per well into 96-well plates for 24 h and infected with ZIKV_PE243_ at a multiplicity of infection (MOI) of 0.01 FFU/cell in the presence of each compound at the established non-cytotoxic concentration. After 72 h, the cells were fixed with 4% formaldehyde, incubated for 30 min, washed with PBS, and the BB was added for the immunofluorescence assay for FFU determination. Viral replication was calculated according to the equation (T/C) × 100%, where T and C represent the FFU mean of the treated group and vehicle control group, respectively.

Data were analyzed for normal distribution to demonstrate the applicability of a parametric or nonparametric tests. Subsequently, two-way ANOVA, using GraphPad Prism 10.3.0 software, was employed to compare the treatment of each compound with DMSO (0.1%) as a negative control, with significance set at *p* < 0.05 [55].

All the ZIKV infection assays were performed at a BSL-2 laboratory under the authorization number CBQ: 163/02 and process SEI: 01.245.006267/2022–14 from the CTNBio—National Technical Commission for Biosecurity from Brazil.

### 3.8. Determination of Antioxidant Potential

#### 3.8.1. DPPH Radical Scavenging Capacity

The experiments were conducted following the methodology of Samaniego-Sánchez et al. (2011) with adaptations by Mar et al. (2021) [56,57]. The free-radical scavenging capacity of *E. punicifolia* leaf extracts was assessed using the DPPH^•^ radical method. A 100 μM methanolic DPPH^•^ solution was prepared. The samples were then prepared at a concentration of 1.0 mg mL^−1^ and mixed with 1900 μL of the methanolic DPPH^•^ solution. Trolox, at a concentration from 100 to 2000, was used as a positive control. The mixture was incubated in the dark at 25 °C for 30 min. Absorbance was measured at 515 nm using a microplate reader (Bio Tek Instruments Inc., Winooski, VT, USA). The antioxidant capacity was quantified in Trolox equivalents, and the assay was performed in triplicate. The relationship between absorbance and Trolox concentration was determined as y = −0.0004x + 0.731, with an R^2^ value of 0.9944. All measurements were made in triplicate, and the results were expressed in micromolar Trolox equivalents (μM Trolox mL^−1^).

#### 3.8.2. ABTS Radical Cation Scavenging Capacity

The ABTS^•+^-scavenging assay involves observing the color bleaching of the ABTS^•+^ solution in the presence of antioxidant extracts at a concentration of 1.0 mg mL^−1^. The methodology of Samaniego-Sánchez et al. (2011) was utilized, with adaptations (Mar et al., 2021) [56,57]. After a 6 min reaction between the sample and the radical at a 1:10 ratio, absorbances were measured at 750 nm using a microplate reader (Bio Tek Instruments Inc., Winooski, VT, USA). Trolox was used to construct the standard curve (y = 0.0003x + 0.7473, R^2^ = 0.999), and the results were expressed as mean ± standard deviation (*n* = 3) of the micromolar Trolox equivalents (μM Trolox mL^−1^).

### 3.9. Antiglycation Activity: Non-Oxidative Pathway

The ability to inhibit the formation of advanced-glycation end-products (AGEs) was evaluated according to the method of Kiho et al. (2004), with slight modifications [58]. The reaction was carried out in triplicate using the following concentrations: 10.0 mg mL^−1^ albumin (BSA), 30 mM fructose, and 1.00 mg mL^−1^ of the sample (dissolved in DMSO). The fructose and BSA solutions were prepared in a phosphate buffer (0.20 M, pH 7.4) with 3.0 mM sodium azide as an antimicrobial agent. The 300 μL of the total reaction mixture consisted of BSA (135 μL), fructose (135 μL), and DMSO or sample (30 μL). The mixture was incubated at 37 °C for 48 h under sterile conditions and in the dark. After incubation, each sample was analyzed in a microplate reader for fluorescence intensity (emission λ 330 nm and excitation λ 420 nm). Aminoguanidine was used as a standard, and DMSO served as a negative control. The percentage inhibition was calculated using Equation (2), and the results are expressed as mean % inhibition ± standard deviation (*n* = 3).

% inhibition = 100 − [Fluora/p/FLuorC] × 100(2)

where: Fluora/p = (White fluorescence − Sample fluorescence); FLuorC = (White fluorescence − Control fluorescence).

### 3.10. Canonical Correlation Analysis (CCA)

CCA was conducted using Pearson’s correlation coefficient as a correlation index between the chemical composition and the bioactivities of the extracts obtained during the dry and rainy periods. The concentrations of the compounds determined using ^1^H NMR, along with the antioxidant potential and AGE inhibition values, were used as variables. Pearson correlation coefficients were calculated using GraphPad Prism 10.3.0 software, with a 95% confidence interval and significance set at *p* < 0.05 (two-tailed) [55].

## 4. Conclusions

This investigation revealed that the extraction system and seasonality significantly influence the quantitative chemical profile and the antioxidant, antiglycation, and antiviral activities of *Eugenia punicifolia* leaf extracts. The MEW (methanol/ethanol/water) system proved to be the most efficient for extracting bioactive compounds, showing strong antioxidant and antiglycation potentials, especially in samples collected during the rainy season. The use of quantitative NMR and canonical correlation analysis (CCA) allowed for the identification and quantification of key phenolic compounds, including gallic acid, catechin, quercetin, and myricetin, which were linked to the observed pharmacological effects. These compounds can serve as chemical markers for tracking the antioxidant, antiglycation, and antiviral activities of *E. punicifolia* leaf extracts. Overall, this research highlights the importance of choosing the appropriate extraction methods and season to maximize the pharmacological potential of plant-based extracts.

## Figures and Tables

**Table 1:** Yields of *E. punicifolia* leaf extracts according to the extraction system and collection period.

Sample	MEW (mg g^−1^ Dry Extract)	M (mg g^−1^ Dry Extract)	EM (mg g^−1^ Dry Extract)	E (mg g^−1^ Dry Extract)
Dry	312.2 ± 13.5 ^ab^	275.8 ± 5.9 ^c^	207.4 ± 4.7 ^e^	117.0 ± 8.9 ^f^
Transition	334.1 ± 7.3 ^a^	298.9 ± 8.6 ^b^	224.9 ± 1.9 ^ed^	118.9 ± 7.8 ^f^
Rainy	328.2 ± 8.1 ^a^	304.2 ± 1.9 ^b^	242.9 ± 9.1 ^d^	124.4 ± 7.1 ^f^

^a, b, c, d, e, f^ Clustering for extraction yield using Tukey’s test and a 95% confidence interval. Extract acronyms: MEW—methanol/ethanol/water; M—methanol; EM—ethanol/methanol; and E—ethanol.

**Table 2:** Scavenging capacity of the DPPH^•^ free-radical and the ABTS^•+^ cation radical expressed in μM TE g^−1^.

Sample	Dry		Transition		Rainy	
	DPPH^•^	ABTS^•+^	DPPH^•^	ABTS^•+^	DPPH^•^	ABTS^•+^
MEW	1317.5 ± 6.6 ^a^	1848.8 ± 6.9 ^a^	1449.2 ± 8.8 ^a^	2008.8 ± 8.4 ^a^	1530.8 ± 5.2 ^a^	2121.0 ± 6.7 ^a^
EM	1139.2 ± 10.1 ^b^	1702.1 ± 10.7 ^b^	1213.3 ± 8.0 ^b^	1766.5 ± 6.9 ^b^	1343.33 ± 8.0 ^b^	1919.9 ± 8.4 ^b^
E	1115.0 ± 9.0 ^c^	1685.4 ± 6.9 ^b^	1189.2 ± 8.8 ^c^	1751.0 ± 6.7 ^b^	1318.3 ± 6.3 ^c^	1827.7 ± 6.7 ^d^
M	1025.8 ± 5.2 ^d^	1645.4 ± 8.4 ^c^	1085.8 ± 3.8 ^d^	1703.2 ± 10.2 ^a^	1140.8 ± 7.6 ^d^	1878.8 ± 8.4 ^c^

^a, b, c, d^ Clustering for scavenging capacity using ANOVA (Tukey’s test and 95% confidence interval). Extract acronyms: MEW—methanol/ethanol/water; M—methanol; EM—ethanol/methanol; and E—ethanol.

**Table 3:** Inhibitory capacity of *E. punicifolia* leaf extracts on the formation of advanced-glycation end-products via the non-oxidative pathway.

Sample	MEW (% Inhibition of AGEs)	M (% Inhibition of AGEs)	EM (% Inhibition of AGEs)	E (% Inhibition of AGEs)
Dry	80.5 ± 1.4 ^b^	90.1 ± 2.0 ^a^	80.4 ± 3.0 ^b^	76.0 ± 1.8 ^b^
Transition	94.3 ± 1.5 ^a^	82.4 ± 2.4 ^b^	83.4 ± 1.6 ^b^	94.0 ± 3.0 ^a^
Rainy	93.1 ± 3.7 ^a^	88.2 ± 3.8 ^ab^	94.8 ± 1.4 ^a^	87.5 ± 3.0 ^a^

^a, b^ Clustering for inhibition of AGEs using ANOVA with Tukey’s test and 95% confidence interval. Extract acronyms: MEW—methanol/ethanol/water; M—methanol; EM—ethanol/methanol; and E—ethanol.

**Table 4:** ^1^H NMR quantification of the main phenolic compounds of *E. punicifolia* leaf extracts using PULCON.

Sample	Quercetin (mg g^−1^ Dry Extract)	Myricetin (mg g^−1^ Dry Extract)	Gallic Acid (mg g^−1^ Dry Extract)	Catechin (mg g^−1^ Dry Extract)	Epigallocatechin (mg g^−1^ Dry Extract)	Sum of Total Phenolics (mg g^−1^ Dry Extract)
E—Dry	1.94 ± 0.01	1.40 ± 0.00	3.49 ± 0.01	5.02 ± 0.01	3.34 ± 0.01	15.24 ± 0.20
E—Transition	2.23 ± 0.01	1.62 ± 0.00	3.54 ± 0.01	4.65 ± 0.02	3.63 ± 0.02	15.75 ± 0.31
E—Rainy	2.23 ± 0.00	1.69 ± 0.00	3.50 ± 0.01	4.54 ± 0.01	3.55 ± 0.01	15.60 ± 0.17
EM—Dry	1.24 ± 0.01	1.45 ± 0.01	3.23 ± 0.01	5.50 ± 0.02	3.60 ± 0.02	15.09 ± 0.37
EM—Transition	1.73 ± 0.00	1.71 ± 0.00	4.04 ± 0.01	6.55 ± 0.01	5.01 ± 0.00	19.13 ± 0.18
EM—Rainy	2.44 ± 0.00	1.87 ± 0.00	3.61 ± 0.01	5.78 ± 0.01	4.47 ± 0.00	18.27 ± 0.07
M—Dry	1.47 ± 0.00	1.75 ± 0.01	3.85 ± 0.02	6.54 ± 0.03	4.28 ± 0.02	17.98 ± 0.43
M—Transition	2.07 ± 0.00	1.60 ± 0.00	4.00 ± 0.02	6.35 ± 0.01	5.55 ± 0.01	19.65 ± 0.18
M—Rainy	2.29 ± 0.01	1.83 ± 0.01	3.91 ± 0.00	5.89 ± 0.02	5.08 ± 0.01	19.11 ± 0.43
MEW—Dry	2.89 ± 0.01	2.13 ± 0.00	3.38 ± 0.03	6.97 ± 0.03	4.87 ± 0.03	20.35 ± 0.35
MEW—Transition	2.70 ± 0.01	1.56 ± 0.01	3.91 ± 0.01	6.67 ± 0.03	5.85 ± 0.02	20.94 ± 0.56
MEW—Rainy	3.16 ± 0.00	1.98 ± 0.00	3.79 ± 0.02	6.12 ± 0.01	5.07 ± 0.01	20.22 ± 0.27

Extract acronyms: MEW—methanol/ethanol/water; M—methanol; EM—ethanol/methanol; and E—ethanol.

## Data Availability

The primary data and contributions of this study are provided within the article and supplementary materials. For additional information, please contact the corresponding author(s).

## Supplementary Materials

The following supporting information can be downloaded at https://www.mdpi.com/article/10.3390/molecules30030713/s1: Figure S1. Effect of extract of *Eugenia punicifolia* leaves on viability of Vero E6 cells. Vero E6 cells were treated with each compound at the highest non-cytotoxic concentration. After 72 h, cell viability was measured via the MTT assay. Viability was measured by absorbance (560 ηM). DMSO was used as the untreated control. Mean values of two independent experiments, each measured in triplicate including the standard error of the mean, are shown. *p* values < 0.05 were considered significant. (**) *p* < 0.01, (***) *p* < 0.001, and (****) *p* < 0.0001, Figure S2. ^1^H NMR spectrum (500 MHz, DMSO-*d*_6_) of MEW extract of *Eugenia punicifolia* leaves collected during the dry season (−1.00 to 10.00 ppm), Figure S3. HSQC spectrum (500 MHz, DMSO-*d*_6_) of MEW extract of *Eugenia punicifolia* leaves collected during the dry season (range magnification of ^1^H: −0.50 to 8.30 ppm—13C: −3.0 to 158.0 ppm), Figure S4. HSQC spectrum (^1^H: 500 MHz, ^13^C: 125 MHz DMSO-*d*_6_) of MEW extract of *Eugenia punicifolia* leaves collected during the dry season (range magnification of ^1^H: 5.50 to 7.80 ppm—^13^C: 96.0 to 128.0 ppm). Signals corresponding to gallic acid (**1**: δ 6.95—δ 108.6), quercetin (**2**: δ 7.30—δ 115.5), myricetin (**3**: δ 7.01—δ 108.7), catechin (**4**: δ 5.93—δ 95.4) and epigallocatechin (**5**: δ 5.89—δ 95.1)], Figure S5. HMBC spectrum (^1^H: 500 MHz, ^13^C: 125 MHz DMSO-*d*_6_) of MEW extract of *Eugenia punicifolia* leaves collected during the dry season (range magnification of ^1^H: −0.50 to 8.50 ppm—13C: −4.7 to 193.3 ppm), Figure S6. HMBC spectrum (^1^H: 500 MHz, ^13^C: 125 MHz DMSO-*d*_6_) of MEW extract of *Eugenia punicifolia* leaves collected during the dry season (range magnification of ^1^H: 5.55 to 7.40 ppm—^13^C: 50.7 to 180.2 ppm), Figure S7. ^1^H NMR spectrum (500 MHz, DMSO-*d*_6_) of extracts of *Eugenia punicifolia* leaves collected during the dry season (−1.00 to 10.00 ppm), Figure S8. ^1^H NMR spectrum (500 MHz, DMSO-*d*_6_—range magnification from 8.00 to 5.50 ppm) of extracts of *Eugenia punicifolia* leaves collected during the dry season. Signals corresponding to gallic acid (**1**: δ 6.95), quercetin (**2**: δ 7.30), myricetin (**3**: δ 7.01), catechin (**4**: δ 5.93) and epigallocatechin (**5**: δ 5.89)]. Extract acronyms: MEW—methanol/ethanol/water; M—methanol; EM—ethanol/methanol; and E—ethanol, Figure S9. ^1^H NMR spectrum (500 MHz, DMSO-*d*_6_) of extracts of *Eugenia punicifolia* leaves collected during the transition season (−1.00 to 10.00 ppm region), Figure S10. ^1^H NMR spectrum (500 MHz, DMSO-*d*_6_—range magnification from 8.00 to 5.50 ppm) of extracts of *Eugenia punicifolia* leaves collected during the transition season. Signals corresponding to gallic acid (**1**: δ 6.95), quercetin (**2**: δ 7.30), myricetin (**3**: δ 7.01), catechin (**4**: δ 5.93) and epigallocatechin (**5**: δ 5.89)]. Extract acronyms: MEW—methanol/ethanol/water; M—methanol; EM—ethanol/methanol; and E—ethanol, Figure S11. ^1^H NMR spectrum (500 MHz, DMSO-*d*_6_) of extracts of *Eugenia punicifolia* leaves collected during the rainy season (−1.00 to 10.00 ppm), Figure S12. ^1^H NMR spectrum (500 MHz, DMSO-*d*_6_—range magnification from 8.00 to 5.50 ppm) of extracts of *Eugenia punicifolia* collected during the transition season. Signals corresponding to gallic acid (**1**: δ 6.95), quercetin (**2**: δ 7.30), myricetin (**3**: δ 7.01), catechin (**4**: δ 5.93) and epigallocatechin (**5**: δ 5.89). Extract acronyms: MEW—methanol/ethanol/water; M—methanol; EM—ethanol/methanol; and E—ethanol, Table S1. Pearson correlation coefficient between the concentration determined by ^1^H NMR and the scavenging capacity of the DPPH radical, the ABTS radical cation and inhibition of AGE formation. **1**—gallic acid, **2**—epigallocatechin, **3**—catechin, **4**—quercetin and **5**—myricetin.
