# Supplementary material for: Investigation of the Influence of the Extraction System and Seasonality on the Pharmacological Potential of Eugenia punicifolia Leaves
Source: Molecules. 2025 Feb 5;30(3):713. doi: 10.3390/molecules30030713 (PMC11820027; doi:10.3390/molecules30030713)
Supplement: Supplementary file 1 [file molecules-30-00713-s001.zip › molecules-3379252-supplementary.pdf]

Supplementary Information

Figure S1. Effect of extract of *Eugenia punicifolia* leaves on viability of Vero E6 cells. Vero E6 cells were treated with each compound at the highest non-cytotoxic concentration. After 72 h, cell viability was measured via the MTT assay. Viability was measured by absorbance (560 nm). DMSO was used as the untreated control. Mean values of two independent experiments, each measured in triplicate including the standard error of the mean, are shown. P values < 0.05 were considered significant. (**) $p < 0.01$, (***) $p < 0.001$, and (****) $p < 0.0001$.

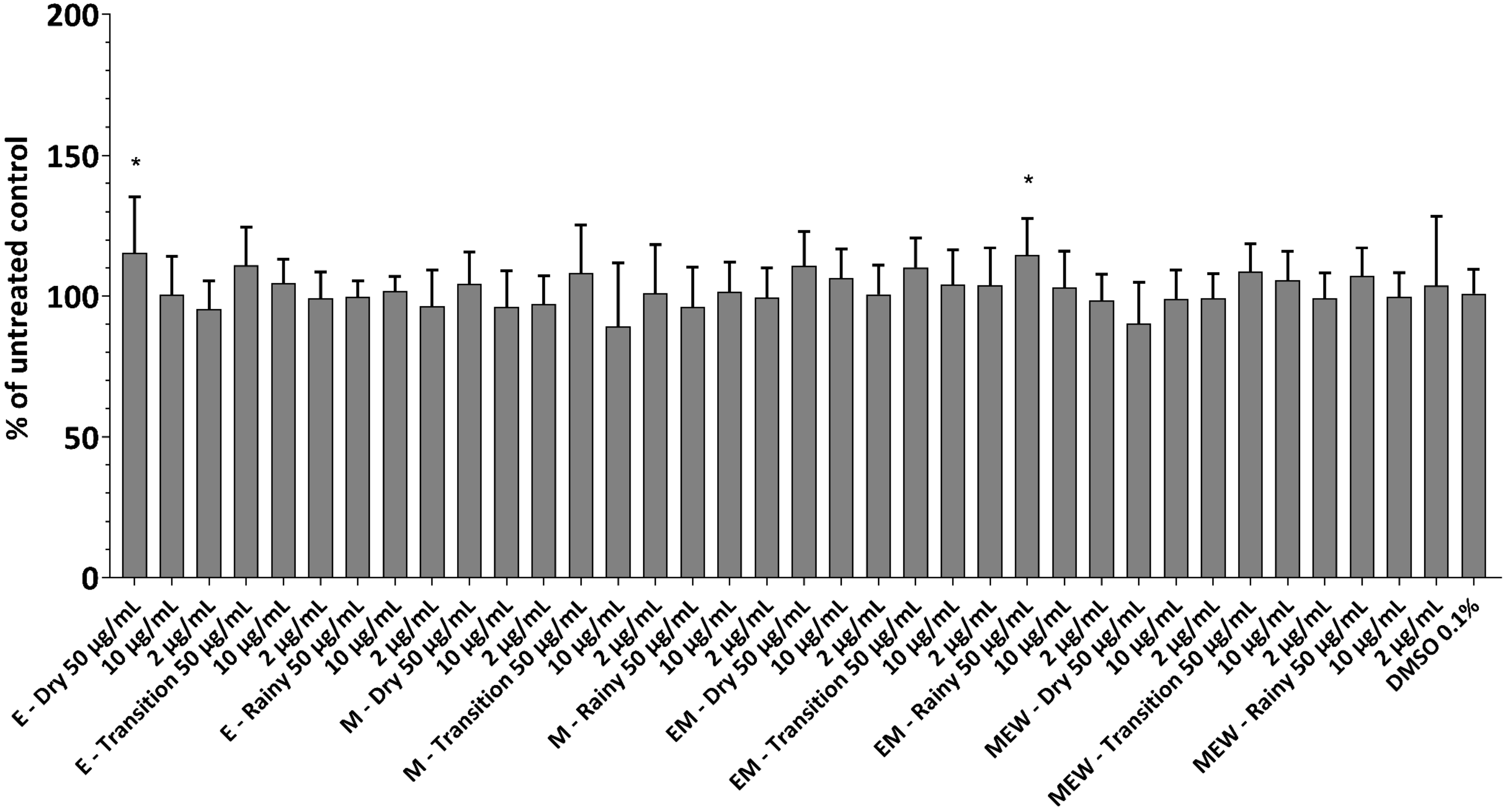
200
150
100
50
0
% of untreated control
*
*
E - Dry 50 µg/mL
10 µg/mL
2 µg/mL
E - Transition 50 µg/mL
10 µg/mL
2 µg/mL
E - Rainy 50 µg/mL
10 µg/mL
2 µg/mL
M - Dry 50 µg/mL
10 µg/mL
2 µg/mL
M - Transition 50 µg/mL
10 µg/mL
2 µg/mL
M - Rainy 50 µg/mL
10 µg/mL
2 µg/mL
EM - Dry 50 µg/mL
10 µg/mL
2 µg/mL
EM - Transition 50 µg/mL
10 µg/mL
2 µg/mL
EM - Rainy 50 µg/mL
10 µg/mL
2 µg/mL
MEW - Dry 50 µg/mL
10 µg/mL
2 µg/mL
MEW - Transition 50 µg/mL
10 µg/mL
2 µg/mL
MEW - Rainy 50 µg/mL
10 µg/mL
2 µg/mL
DMSO 0.1%

Figure S2. ^{1}H NMR spectrum (500 MHz, DMSO-d_6) of MEW extract of *Eugenia punicifolia* leaves collected during the dry season (– 1.00 to 10.00 ppm).

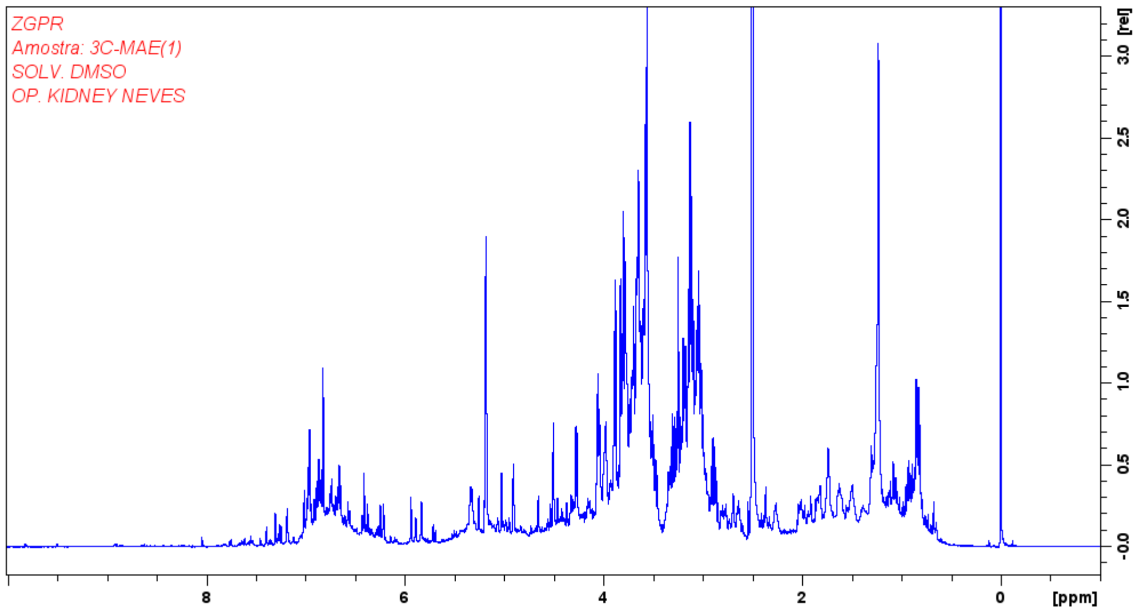

Figure S3. HSQC spectrum (500 MHz, DMSO-d_6) of MEW extract of *Eugenia punicifolia* leaves collected during the dry season (range magnification of ^{1}H: - 0.50 to 8.30 ppm - 13C: - 3.0 to 158.0 ppm).

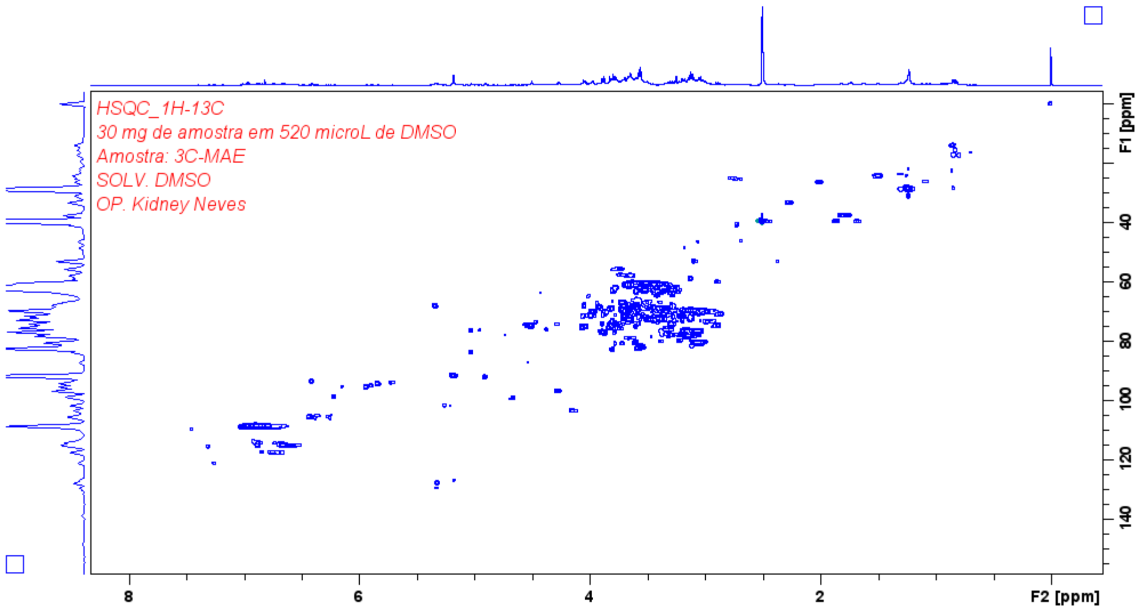

Figure S4. HSQC spectrum (^{1}H: 500 MHz, ^{13}C: 125 MHz DMSO-d_6) of MEW extract of *Eugenia punicifolia* leaves collected during the dry season (range magnification of ^{1}H: 5.50 to 7.80 ppm - ^{13}C: 96.0 to 128.0 ppm). Signals corresponding to gallic acid (**1**: δ 6.95 - δ 108.6), quercetin (**2**: δ 7.30 - δ 115.5), myricetin (**3**: δ 7.01 - δ 108.7), catechin (**4**: δ 5.93 - δ 95.4) and epigallocatechin (**5**: δ 5.89 - δ 95.1)].

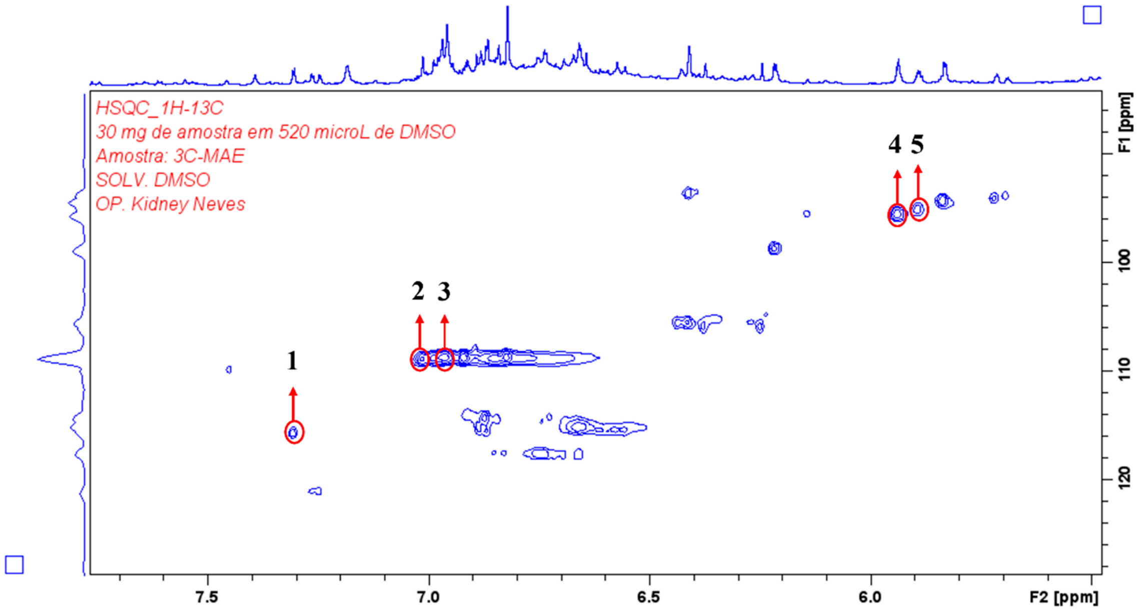

Figure S5. HMBC spectrum (^{1}H: 500 MHz, ^{13}C: 125 MHz DMSO-d_6) of MEW extract of *Eugenia punicifolia* leaves collected during the dry season (range magnification of ^{1}H: - 0.50 to 8.50 ppm - 13C: - 4.7 to 193.3 ppm),.

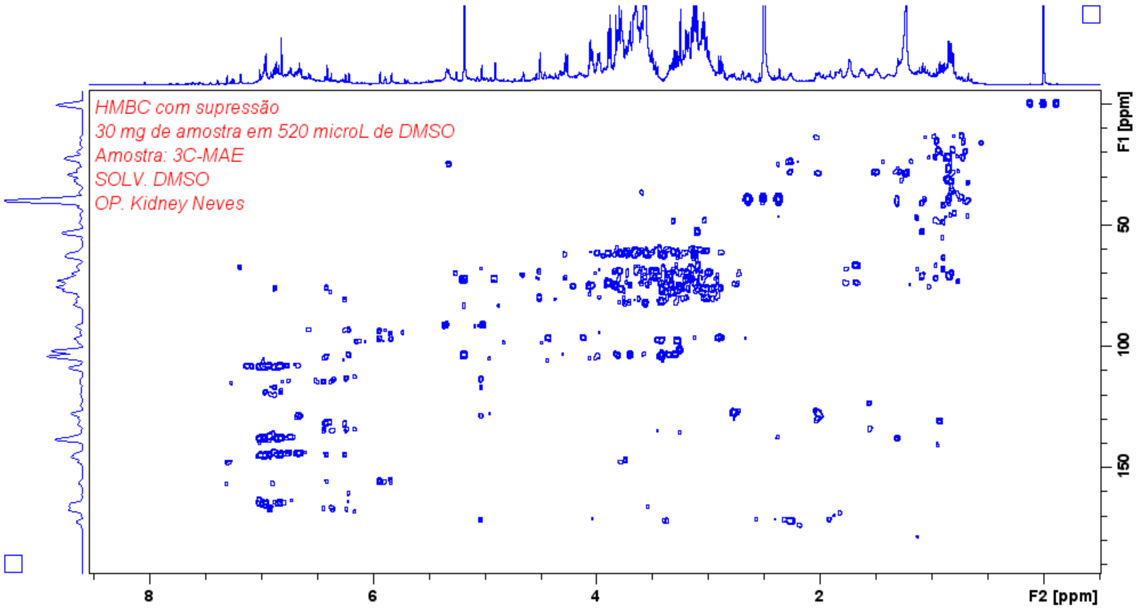

Figure S6. HMBC spectrum (^{1}H: 500 MHz, ^{13}C: 125 MHz DMSO-d_6) of MEW extract of *Eugenia punicifolia* leaves collected during the dry season (range magnification of ^{1}H: 5.55 to 7.40 ppm - ^{13}C: 50.7 to 180.2 ppm).

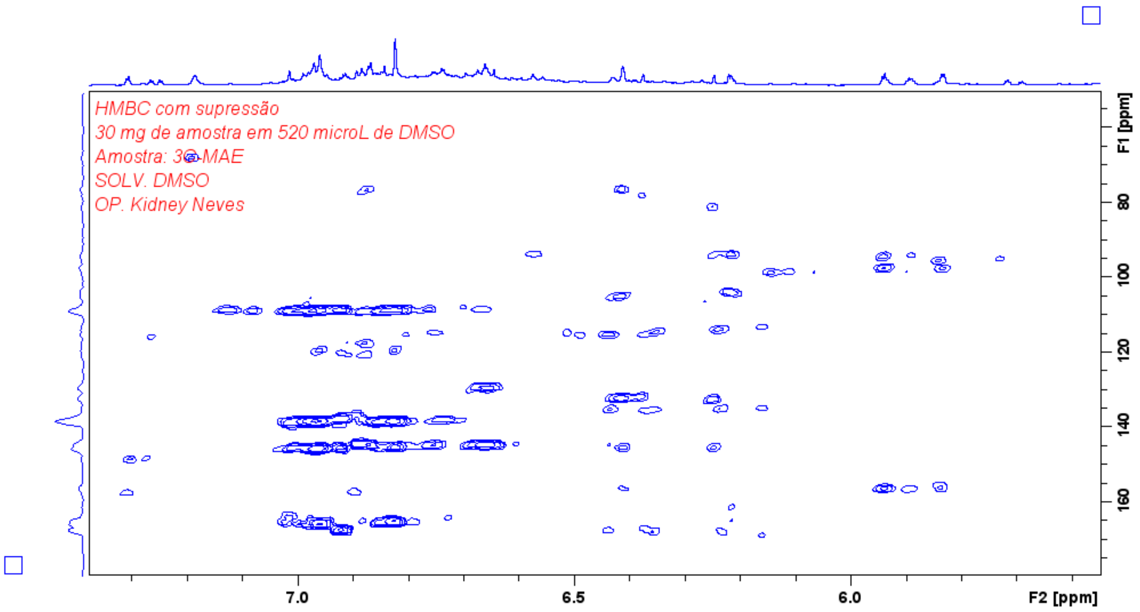

Figure S7. ^{1}H NMR spectrum (500 MHz, DMSO-d_6) of extracts of *Eugenia punicifolia* leaves collected during the dry season (- 1.00 to 10.00 ppm).

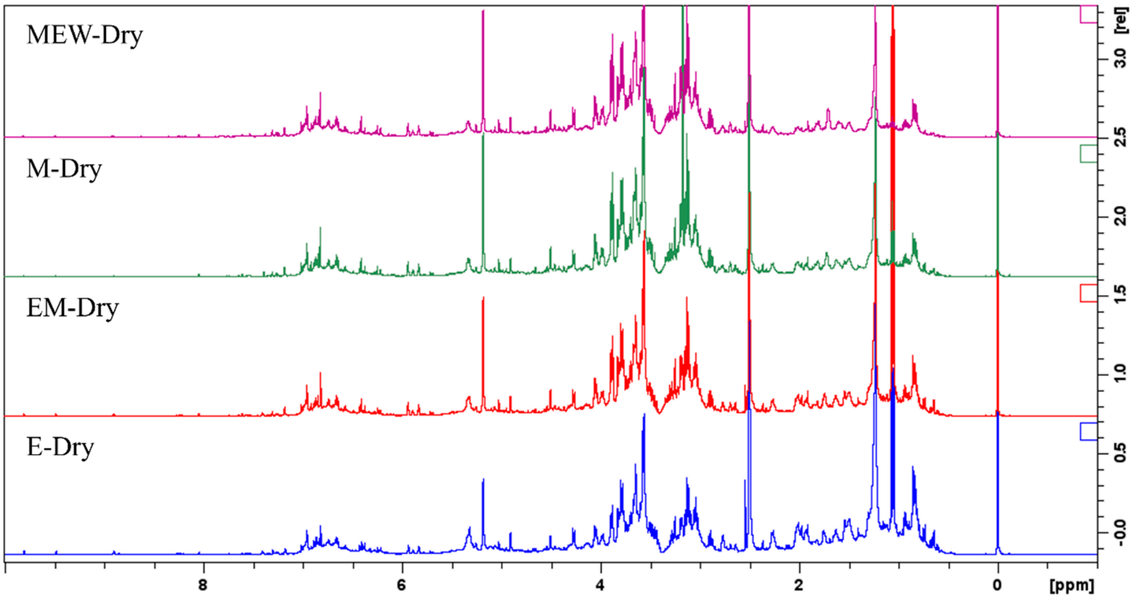

Figure S8. ^{1}H NMR spectrum (500 MHz, DMSO-d_6 - range magnification from 8.00 to 5.50 ppm) of extracts of *Eugenia punicifolia* leaves collected during the dry season. Signals corresponding to gallic acid (**1**: δ 6.95), quercetin (**2**: δ 7.30), myricetin (**3**: δ 7.01), catechin (**4**: δ 5.93) and epigallocatechin (**5**: δ 5.89)]. Extract acronyms: MEW – methanol:ethanol:water; M – methanol; EM – ethanol:methanol; and E – ethanol.

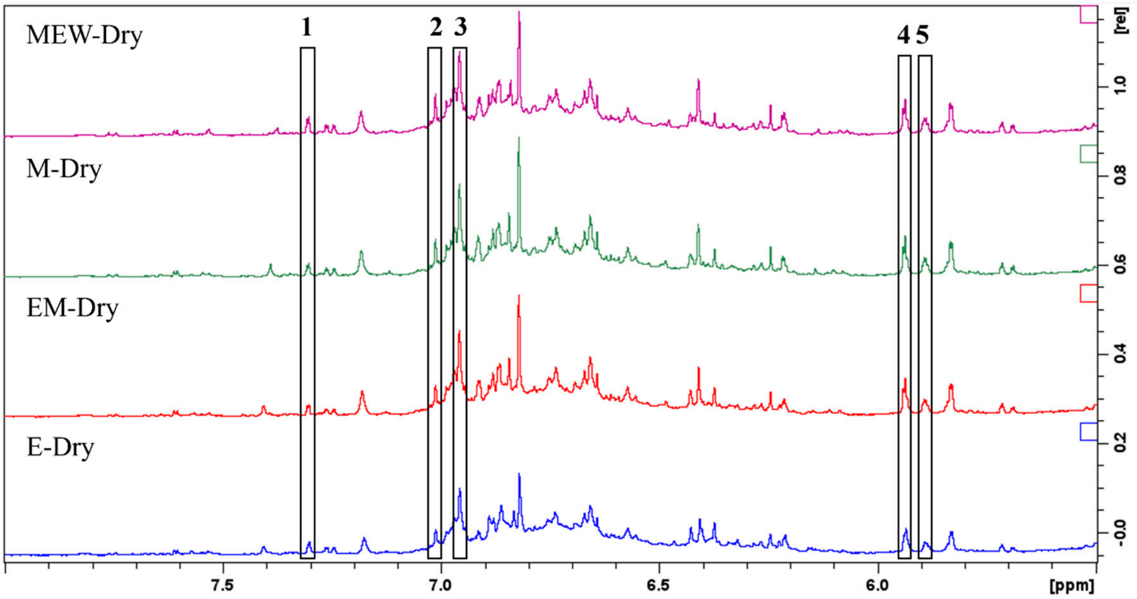

Figure S9. ^{1}H NMR spectrum (500 MHz, DMSO-d_6) of extracts of *Eugenia punicifolia* leaves collected during the transition season (- 1.00 to 10.00 ppm region).

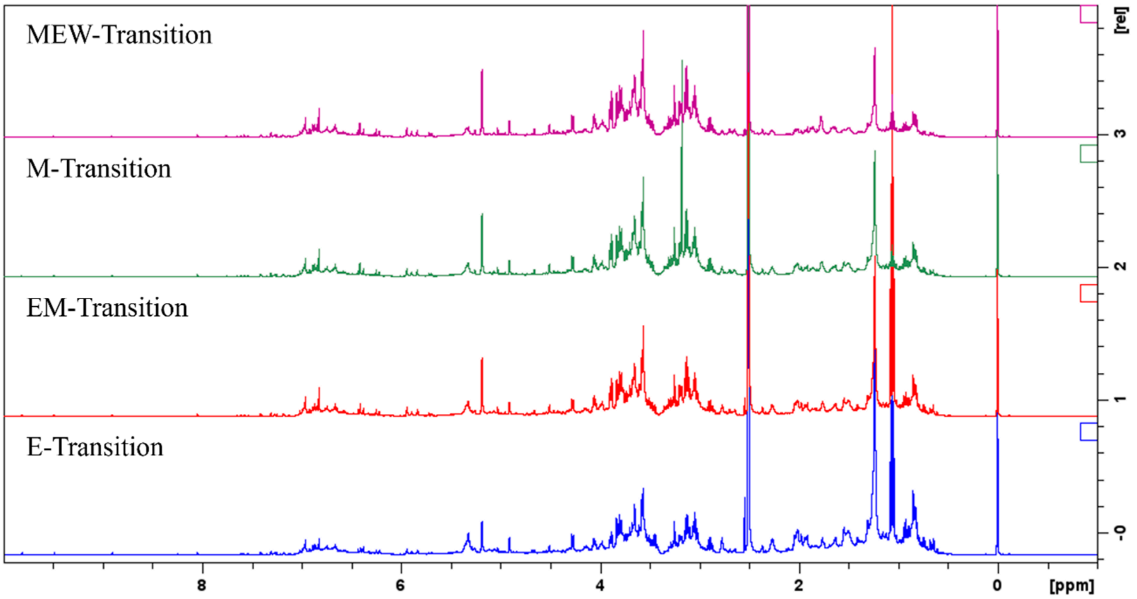

Figure S10. ^{1}H NMR spectrum (500 MHz, DMSO-d_6 - range magnification from 8.00 to 5.50 ppm) of extracts of *Eugenia punicifolia* leaves collected during the transition season. Signals corresponding to gallic acid (**1**: δ 6.95), quercetin (**2**: δ 7.30), myricetin (**3**: δ 7.01), catechin (**4**: δ 5.93) and epigallocatechin (**5**: δ 5.89)]. Extract acronyms: MEW – methanol:ethanol:water; M – methanol; EM – ethanol:methanol; and E – ethanol.

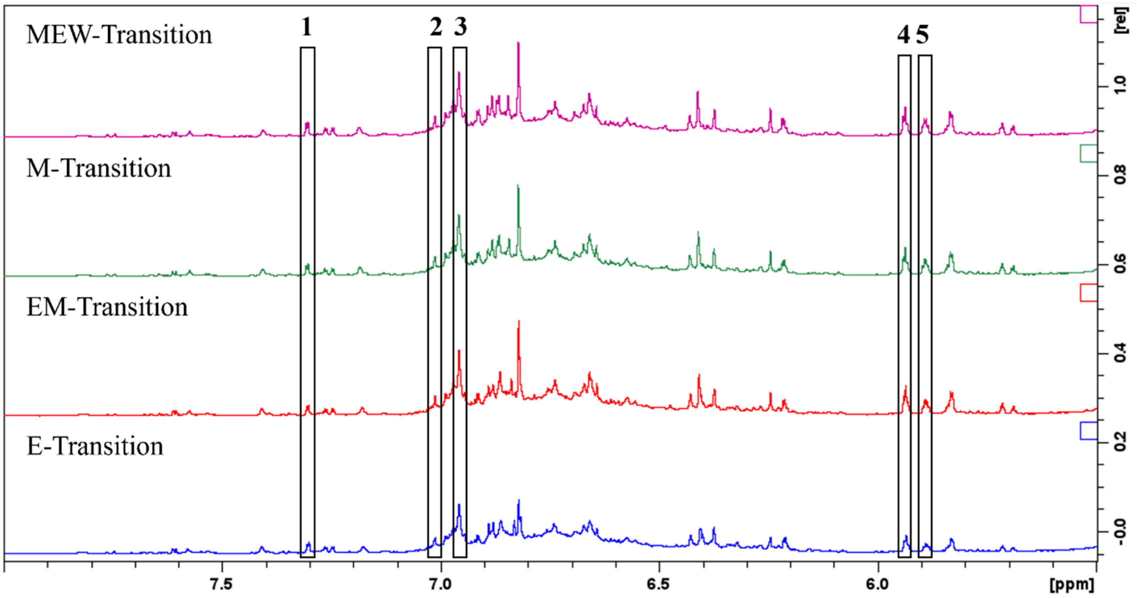

Figure S11. ^{1}H NMR spectrum (500 MHz, DMSO-d_6) of extracts of *Eugenia punicifolia* leaves collected during the rainy season (- 1.00 to 10.00 ppm).

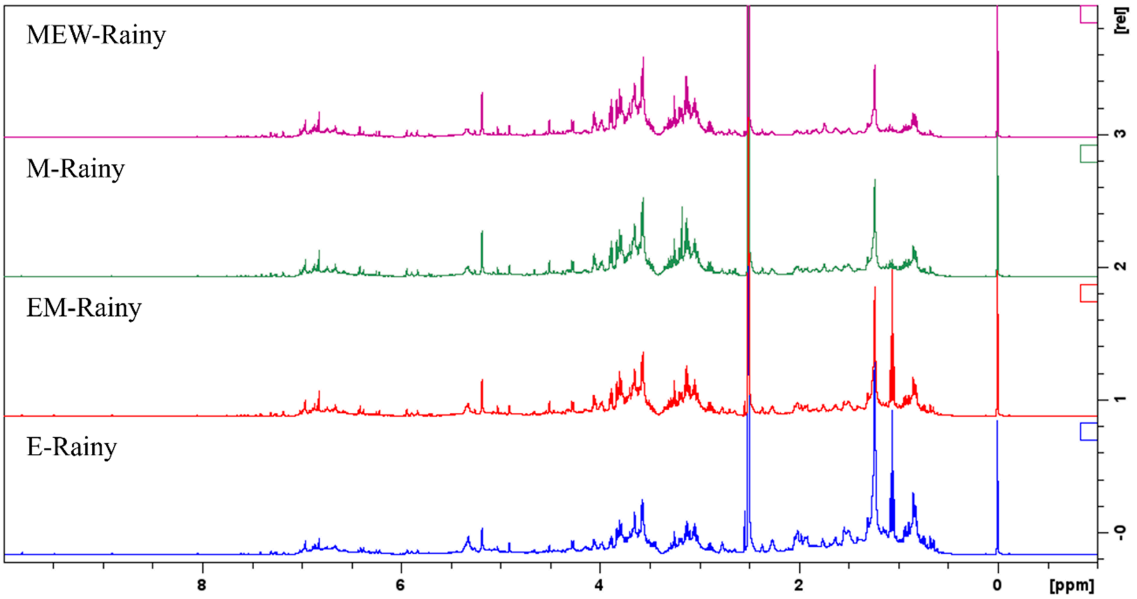

Figure S12. ^{1}H NMR spectrum (500 MHz, DMSO-d_6 - range magnification from 8.00 to 5.50 ppm) of extracts of *Eugenia punicifolia* collected during the transition season. Signals corresponding to gallic acid (**1**: δ 6.95), quercetin (**2**: δ 7.30), myricetin (**3**: δ 7.01), catechin (**4**: δ 5.93) and epigallocatechin (**5**: δ 5.89). Extract acronyms: MEW – methanol:ethanol:water; M – methanol; EM – ethanol:methanol; and E – ethanol.

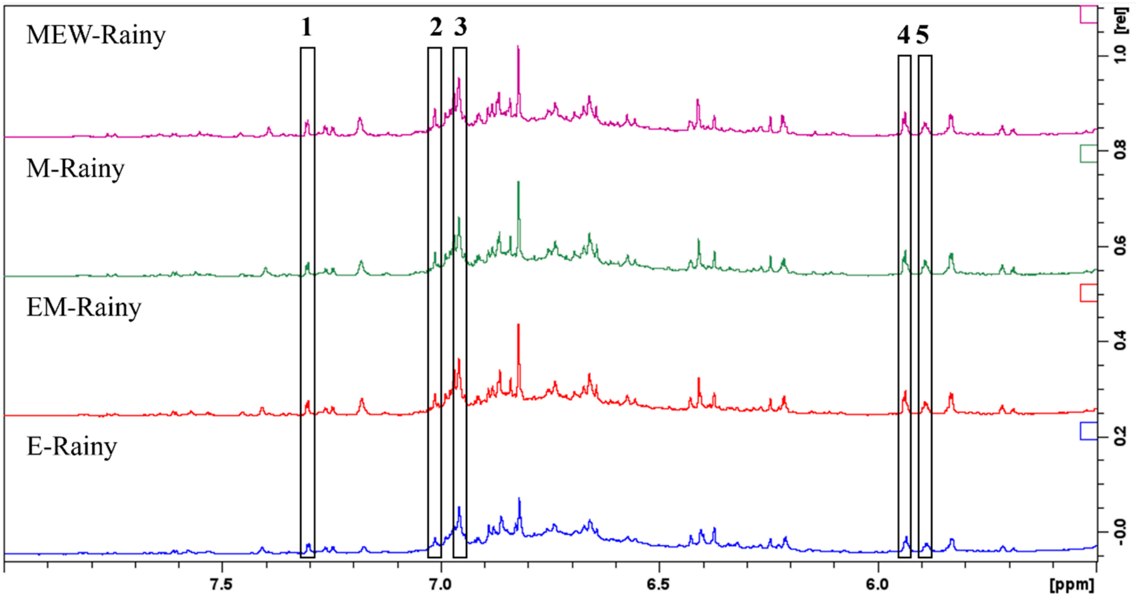

Table S1. Pearson correlation coefficient between the concentration determined by ^{1}H NMR and the scavenging capacity of the DPPH radical, the ABTS radical cation, and inhibition of AGE formation. **1** - gallic acid, **2** - epigallocatechin, **3** - catechin, **4** - quercetin, and **5** - myricetin.

	(**1**)	(**2**)	(**3**)	(**4**)	(**5**)	$DPPH^{\bullet}$	$ABTS^{\bullet +}$	AGEs inhibition
(**1**)	1.00	0.54	0.25	0.22	0.32	− 0.01	0.32	0.60
(**2**)	0.54	1.00	0.73	0.66	0.85	0.41	0.67	0.50
(**3**)	0.25	0.73	1.00	0.29	0.70	0.05	0.16	0.16
(**4**)	0.22	0.66	0.29	1.00	0.78	0.85	0.87	0.33
(**5**)	0.32	0.85	0.70	0.78	1.00	0.58	0.65	0.48
$DPPH^{\bullet}$	− 0.01	0.41	0.05	0.85	0.58	1.00	0.90	0.42
$ABTS^{\bullet +}$	0.32	0.67	0.16	0.87	0.65	0.90	1.00	0.56
AGEs inhibition	0.60	0.50	0.16	0.33	0.48	0.42	0.56	1.00
